# Copper tolerance mediated by polyphosphate degradation and low-affinity inorganic phosphate transport system in *Escherichia coli*

**DOI:** 10.1186/1471-2180-14-72

**Published:** 2014-03-19

**Authors:** Mariana Grillo-Puertas, Lici Ariane Schurig-Briccio, Luisa Rodríguez-Montelongo, María Regina Rintoul, Viviana Andrea Rapisarda

**Affiliations:** 1 Instituto Superior de Investigaciones Biológicas, INSIBIO (CONICET-UNT) and Instituto de Química Biológica “Dr Bernabé Bloj”, Facultad de Bioquímica, Química y Farmacia (UNT), Chacabuco 461 CP T4000ILI, Tucumán, Argentina; 2 Present address: Department of Biochemistry, University of Illinois, Urbana IL61801, USA

**Keywords:** *Escherichia coli*, Copper tolerance, Stationary phase, Polyphosphate, Inorganic phosphate

## Abstract

**Background:**

Metal tolerance in bacteria has been related to polyP in a model in which heavy metals stimulate the polymer hydrolysis, forming metal-phosphate complexes that are exported. As previously described in our laboratory, *Escherichia coli* cells grown in media containing a phosphate concentration >37 mM maintained an unusually high polyphosphate (polyP) level in stationary phase. The aim of the present work was to evaluate the influence of polyP levels as the involvement of low-affinity inorganic phosphate transport (Pit) system in *E. coli* copper tolerance.

**Results:**

PolyP levels were modulated by the media phosphate concentration and/or using mutants in polyP metabolism. Stationary phase wild-type cells grown in high phosphate medium were significantly more tolerant to copper than those grown in sufficient phosphate medium. Copper addition to tolerant cells induced polyP degradation by PPX (an exopolyphosphatase), phosphate efflux and membrane polarization. *ppk*^*−*^*ppx*^*−*^ (unable to synthesize/degrade polyP)*, ppx*^*−*^ (unable to degrade polyP) and Pit system mutants were highly sensitive to metal even in high phosphate media. In exponential phase, CopA and polyP-Pit system would act simultaneously to detoxify the metal or one could be sufficient to safeguard the absence of the other.

**Conclusions:**

Our results support a mechanism for copper detoxification in exponential and stationary phases of *E. coli*, involving Pit system and degradation of polyP. Data reflect the importance of the environmental phosphate concentration in the regulation of the microbial physiological state.

## Background

Inorganic polyphosphate (polyP) is a linear polymer of hundreds of orthophosphate residues linked by phosphoanhydride bonds. The main enzymes associated with polyP metabolism in bacteria are polyphosphate kinase (PPK, encoded by *ppk*) and exopolyphosphatase (PPX, encoded by *ppx*) [1,2]. In most organisms, including bacteria, archaea and eukaryotes, metal tolerance was related to polyP levels [3]. Rachlin *et al*. [4] have proposed that polyP, as a metal chelator, reduces intracellular heavy metals concentration in the Cyanophycean alga *Plectonema boryanum*. Similarly, resistance to cadmium in *Anacystis nidulans* R2 strain [5] and in *Klebsiella aerogenes*[6] was related to high polyP levels. Keasling proposed a model where metals (Na^+^, Mg^2+^, Co^2+^ Cd^2+^) can be chelated by polyP and/or regulate the activity of PPX, which would in turn degrade polyP, allowing the removal of metal-phosphate complexes possibly via low-affinity inorganic phosphate transport (Pit) system [7]. This model was supported in acidophilic bacteria [8] and archaea [9], where Cu^2+^ increases PPX activity and phosphate (Pi) efflux.

Pit system in *Escherichia coli* includes PitA (encoded by *pitA*) and PitB (encoded by *pitB*) [10]. van Veen *et al*. [11] have shown that Pit can reversibly transport Ca^2+^, Co^2+^ or Mg^2+^ phosphates in *E. coli* and *Acinetobacter johnsonii*. The uptake of a neutral metal-phosphate (MeHPO) complex is mediated by an electrogenic proton symport mechanism. Conversely, the excretion of the metal-phosphate complex via Pit generates a proton motive force in *A. johnsonii*[12].

Copper is an essential nutrient required for many biochemical functions, acting as a cofactor for several enzymes [13]. However, copper is also a toxic element able to catalyze free radicals formation, producing alteration of nucleic acids, lipids and proteins [14,15]. Thus, cells ensure their viability by a tight regulation of copper levels, involving several homeostatic mechanisms. *E. coli* is equipped with multiple systems to ensure copper handling under varying environmental conditions. For instance, the Cu^+^-translocating P-type ATPase CopA is responsible for removing excess Cu^+^ from the cytoplasm. Multi-copper oxidase CueO and the multi-component copper transport system CusCFBA appears to safeguard the periplasmic space from copper-induced toxicity [16-18]. In aerobic conditions, *E. coli* usually tolerate copper concentrations in the μM range, although minimal inhibitory concentrations for metals depend on the growth media and the methodology used [17-20].

Stationary phase cells are particularly vulnerable to oxidative damage since they lack the energy and materials needed to repair or replace the damaged molecules. In our laboratory, it has been demonstrated that *E. coli* stationary cells presented high viability, low oxidative damage and elevated resistance to exogenous H_2_O_2_ when Pi concentration in the medium was above 37 mM [21]. These events were related to the maintenance of high polyP level in late stationary phase [22].

According to the model proposed previously by Keasling [7], we examined here the involvement of polyP metabolism and Pit system components in *E. coli* copper tolerance in stationary or exponential phase cells. Our approach included the use of mutants in PPK, PPX, PitA and PitB encoding genes and the modulation of polyP levels by varying media phosphate concentration.

## Results

### Cu^2+^ tolerance of stationary phase cells grown in different phosphate concentration media

**Figure 1:**
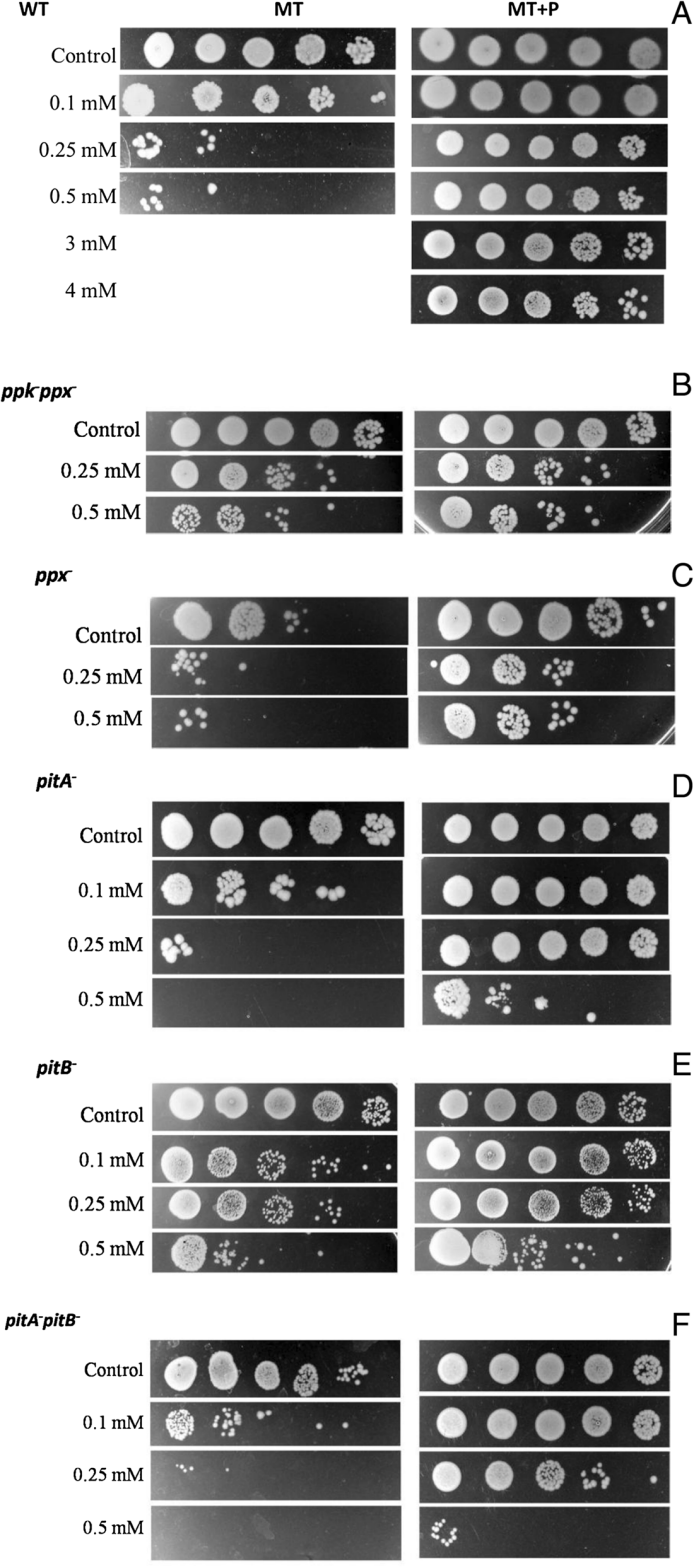
**Copper tolerance in stationary phase cells.** Copper tolerance of 48 h MT or MT + P growing cells of the indicated strains (panels **A**-**F**) was determined after one-hour exposure with different copper concentrations. Serial dilutions of cells incubated without copper (control) or treated cultures were spotted in LB-agar plates. The last spot of each strip was loaded with 1/100000 dilution of original cultures. Data are representative of at least four independent experiments.

The ability to tolerate Cu^2+^ of MC4100 wild-type (WT) cells, grown to stationary phase in media with different phosphate concentration, was evaluated by semiquantitative resistance assay (Figure 1A). Cells grown for 48 h in MT medium (sufficient Pi concentration) were sensitive to 0.25 mM Cu^2+^. Cells grown in MT + P medium (high Pi concentration) tolerated metal concentrations as high as 4 mM. In agreement with previous results [22], Table 1 shows the maintenance of high polyP level in late stationary phase cells grown in MT + P. Differences in tolerance due to media Pi concentration were also observed using LB and LB + P, defined as LB containing 40 mM phosphate buffer pH 7 [23], (data not shown).

**Table 1:** PolyP levels during growth in different Pi concentrations media

	polyP (AU)*											
	**MC4100**		***ppk***^***−***^ ***ppx***^***−***^		***ppx***^***−***^		***pitA***^***−***^ ***pitB***^***−***^		***pitA***^***−***^		***pitB***	
	**MT**	**MT + P**	**MT**	**MT + P**	**MT**	**MT + P**	**MT**	**MT + P**	**MT**	**MT + P**	**MT**	**MT + P**
6 h	123650 ± 10540a	152951 ± 8120a	45541 ± 5563a	38254 ± 4521a	220152 ± 15120a	252651 ± 11120a	80524 ± 9452a	91523 ± 8563a	82536 ± 8652a	95623 ± 9563a	81524 ± 9452a	90523 ± 5563a
24 h	54000 ± 9500b	125420 ± 10245a	42564 ± 4521a	40251 ± 6523a	200536 ± 16245a	241536 ± 12155a	32564 ± 4152b	93056 ± 6652a	24563 ± 3254b	89654 ± 10254a	28564 ± 4152b	88056 ± 8652a
48 h	44652 ± 4556b	138456 ± 8486a	38563 ± 7521a	41251 ± 5125a	208456 ± 12486a	238456 ± 10286a	22563 ± 5634b	89862 ± 4128a	32564 ± 4635b	92365 ± 8365a	20563 ± 5634b	91862 ± 4658a

*Fluorescence 550 nm. For each strain, different letters indicate significant differences among conditions according to Tukey’s test with a *p*-value of 0.05.

As a first step to elucidate the differential copper tolerance in cells grown in MT or MT + P for 48 h, assays using *ppk*^*−*^*ppx*^*−*^ (unable to synthesize/degrade polyP [24,25]) and *ppx*^*−*^ (unable to degrade polyP) cells were performed in these conditions. Both mutants were highly sensitive to metal even in MT + P (Figure 1B and C). Note that, polyP levels in *ppx*^*−*^ strain were always high, independently of the growth phase and the media used, while the *ppkppx* mutant exhibits greatly reduced synthesis of polyP, evidenced by low values of fluorescence emission (Table 1).

The implication of Pit system components in copper tolerance was also analyzed using *E. coli* strains lacking one or both transporter encoding genes (Figure 1D-F). *pitA* and *pitB* single mutants were unable to tolerate 0.5 mM Cu^2+^ in both media. This sensitivity was more pronounced in the *pitApitB* double mutant. It is worth noting that polyP levels in Pit system mutants depended on media Pi concentration, similarly to WT (Table 1).

Above results using different strains and culture media support the idea that stationary phase copper tolerance is mediated by a mechanism which involves both polyP metabolism and Pit system.

### PolyP levels in cells exposed to Cu^2+^

**Figure 2:**
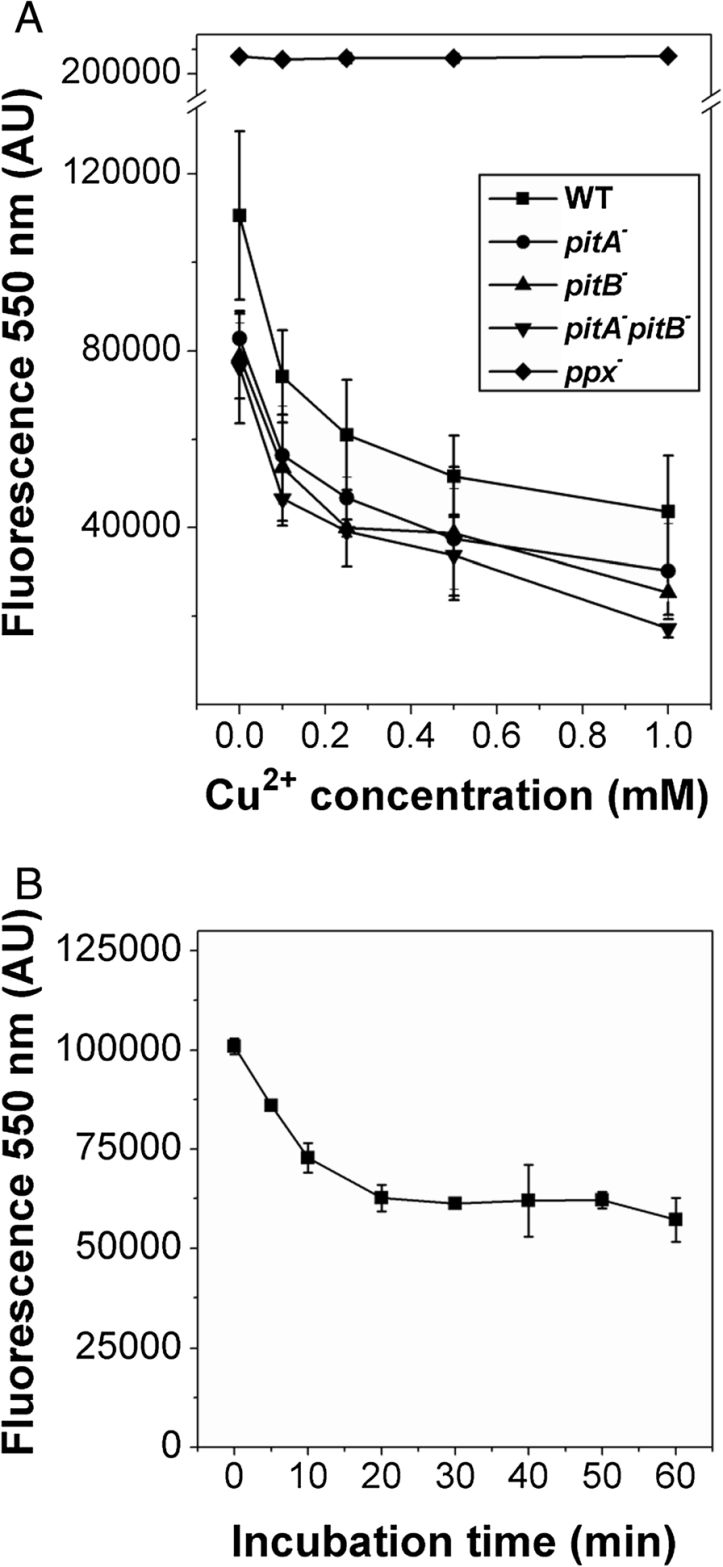
**PolyP levels of stationary phase cells exposed to copper. (A)** Cells of the indicated strains grown in MT + P for 48 h were exposed to increasing copper concentrations for 1 h. After incubations, polyP was quantified as described in Methods using DAPI fluorescence. **(B)** Time-course of polyP degradation in 48 h MT + P WT cells incubated with 0.25 mM Cu^2+^. Data are expressed as average ± SD of five independent experiments. DAPI emission was undetectable in cell free controls with or without copper addition.

Considering that the only presence or abundance of polyP during stationary phase is not sufficient to cope with copper toxicity in our conditions (see *ppx*^*−*^ sensitive phenotype), it might be that copper were responsible to induce degradation of polyP by PPX. In order to explore the occurrence of polymer degradation after metal addition, the effect of different Cu^2+^ concentrations on stationary phase polyP levels was evaluated in MT + P cells (Figure 2A). A copper dependent decrease in polyP levels was observed in WT, *pitA*^*−*^*pitB*^*−*^*, pitA*^*−*^ and *pitB*^*−*^ after one-hour exposure to metal. PolyP degradation induced by copper was dependent on PPX, since metal addition did not affect the polymer levels in *ppx* mutant. PolyP degradation in WT cells took place immediately after copper addition (Figure 2B).

### Pi efflux in cells exposed to Cu^2+^

**Figure 3:**
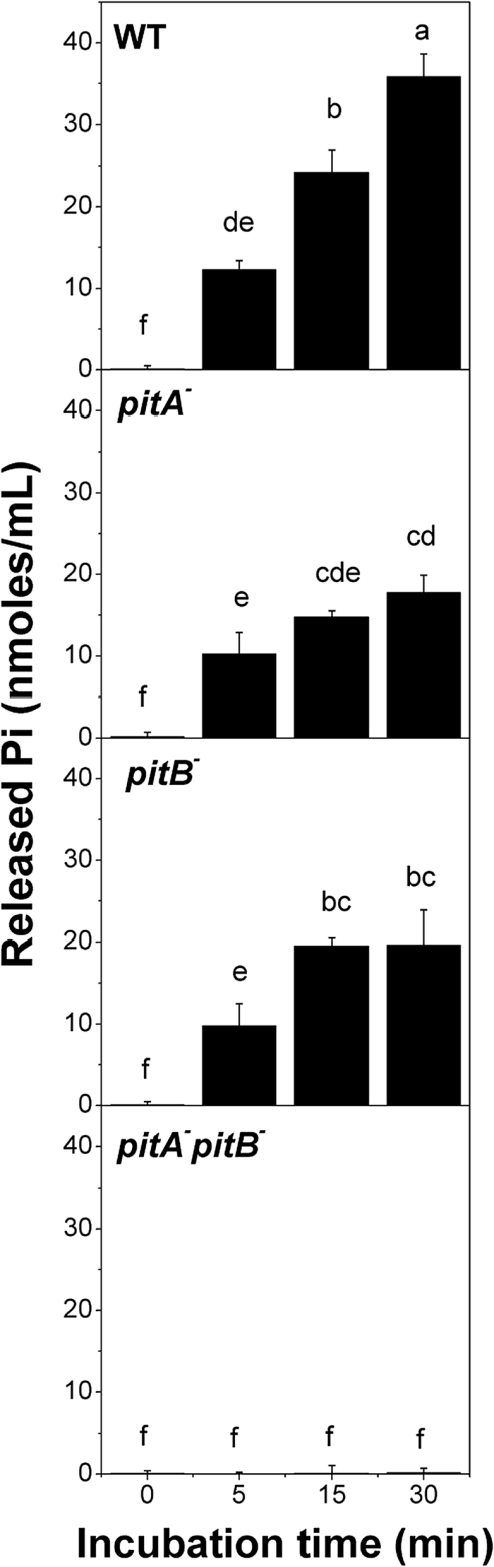
**Pi efflux from stationary phase cells exposed to copper.** 48 h MT + P cells of the indicated strains were resuspended in T buffer and exposed to 0.25 mM Cu^2+^ for the indicated times. Pi was quantified in supernatants as described in Methods. Data are expressed as average ± SD of four independent experiments. Different letters indicate significant differences according to Tukey’s test with a *p*-value of 0.05.

In view of the copper dependent polyP degradation and discarding the chelating effect of the polymer, Pi liberated from the reaction could form complexes with the metal which would be taken out from the cell by Pit system, contributing to detoxify the intracellular environment. Thus, we aimed to test if metal also induces Pi extrusion in stationary phase cells. Time-dependent Pi release was measured in cells exposed to 0.25 mM Cu^2+^. WT cells released around 40 nmoles Pi mL^−1^ at 30 min (Figure 3). For *pitA* and *pitB* single mutants, Pi exported at 30 min was 50% lower than that of WT cells. No Pi release was detected when *pitA*^*−*^*pitB*^*−*^ was used (Figure 3). It is worth noting that Pi was not detected in supernatants of either WT cells incubated without copper or *ppx*^*−*^ cells incubated with copper (data not shown). Viability of all tested strains was maintained after 30 min-exposure to 0.25 mM copper in T buffer (data not shown). Taken together, Pi efflux would be associated to high polyP levels in stationary phase, its degradation in the presence of copper and to the functionality of the Pit system.

### Membrane polarization in cells exposed to Cu^2+^

**Figure 4:**
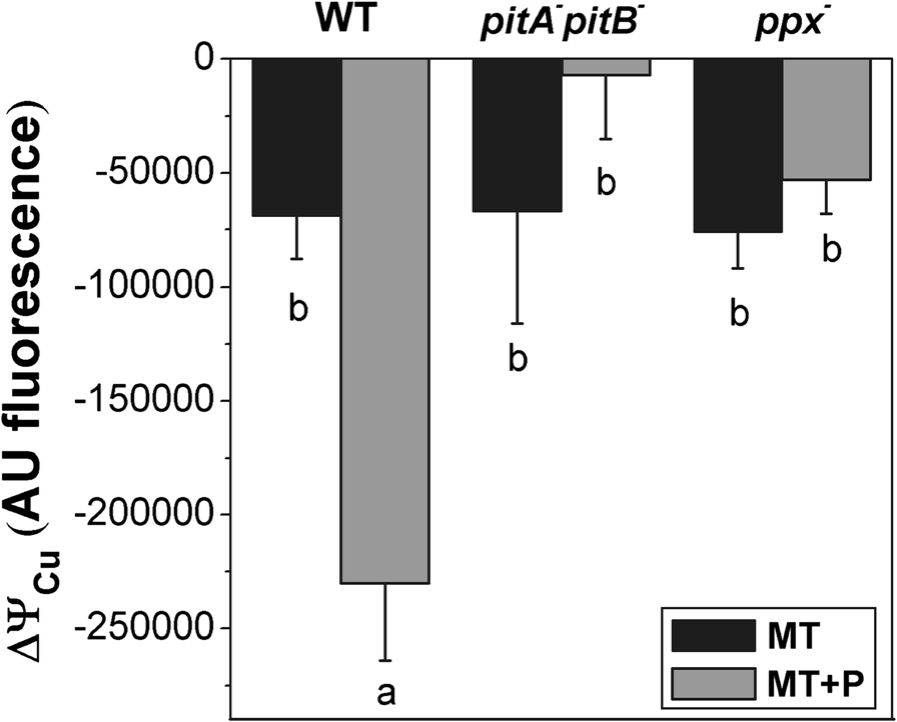
**Membrane potential in stationary phase cells exposed to copper.** 48 h MT or MT + P cells of the indicated strains were resuspended in T buffer and diluted in 5 mM HEPES buffer pH 7.5 to an OD_560nm_ = 0.1. Fluorescence as Arbitrary Units (AU) was measured after addition of the specific dye DisC3[5]. After dye stabilization 0.1 mM Cu^2+^ was added. ΔΨ_Cu_ was the difference between the fluorescence value after 5 min incubation with Cu^2+^ (ΔΨ_f_) and initial stabilization value (ΔΨ_i_). Data are expressed as average ± SD of seven independent experiments. Different letters indicate significant differences according to Tukey’s test with a *p*-value of 0.05.

Since Pit was described as a metal-phosphate:H^+^ symporter [11,26], possible changes in cells membrane potentials after Cu^2+^ addition were studied. Figure 4 shows that copper produced a significant increase in membrane polarization in MT + P WT cells in respect to values of MT WT cells or *pitApitB* and *ppx* mutants in both media. When distillated water was added as a control, no changes in membrane polarization were observed (not shown). These data supported additional evidence indicating that metal-phosphate complexes can be removed from cells via Pit system after copper-dependent polyP degradation.

### Cu^2+^ tolerance of exponential phase cells

**Figure 5:**
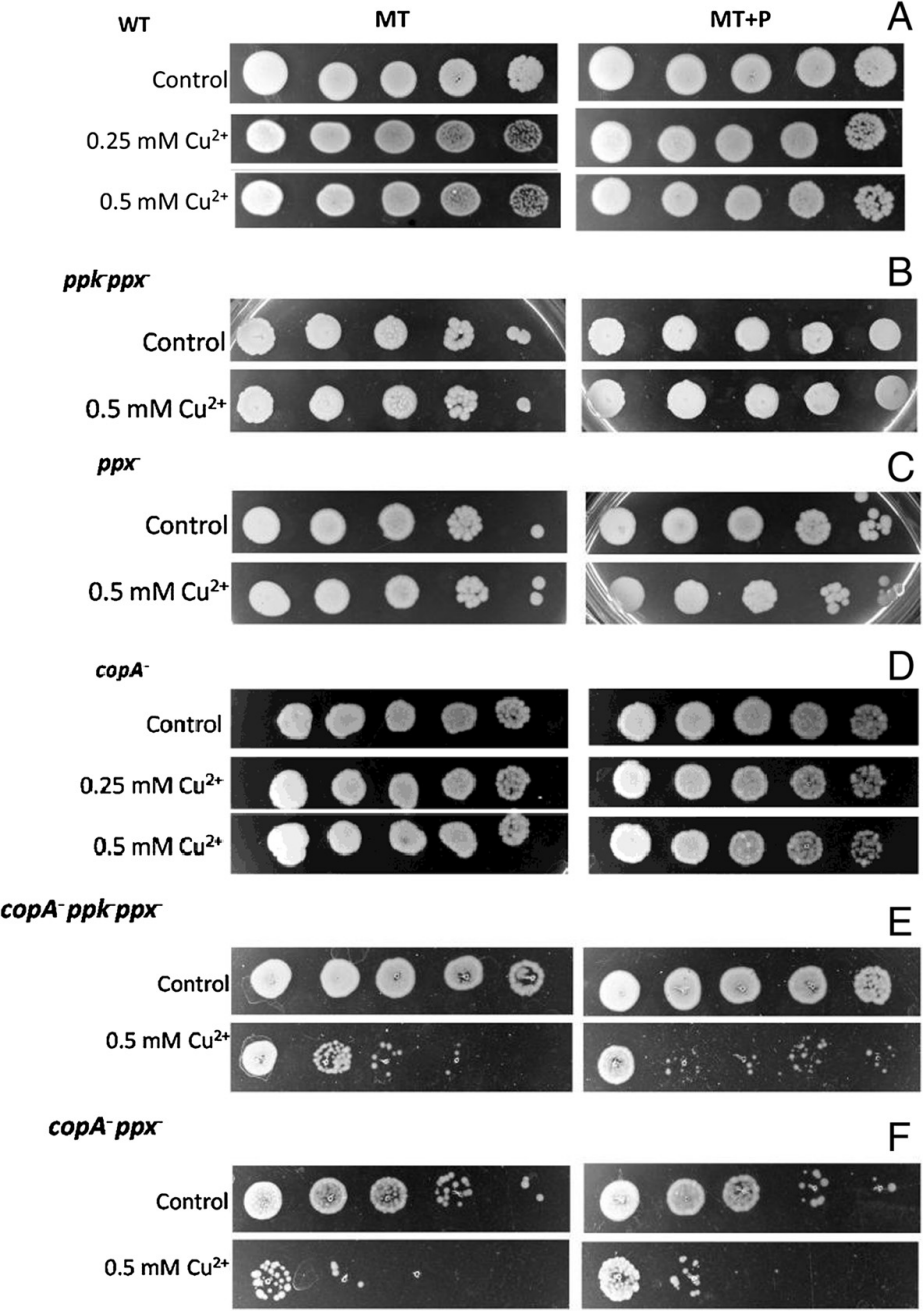
**Copper tolerance in exponential phase cells.** Copper tolerance of 6 h MT or MT + P growing cells of the indicated strains (panels **A**-**F**) was determined after one-hour exposure with different copper concentrations. Serial dilutions of cells incubated without copper (control) or treated cultures were spotted in LB-agar plates. Data are representative of at least four independent experiments.

**Figure 6:**
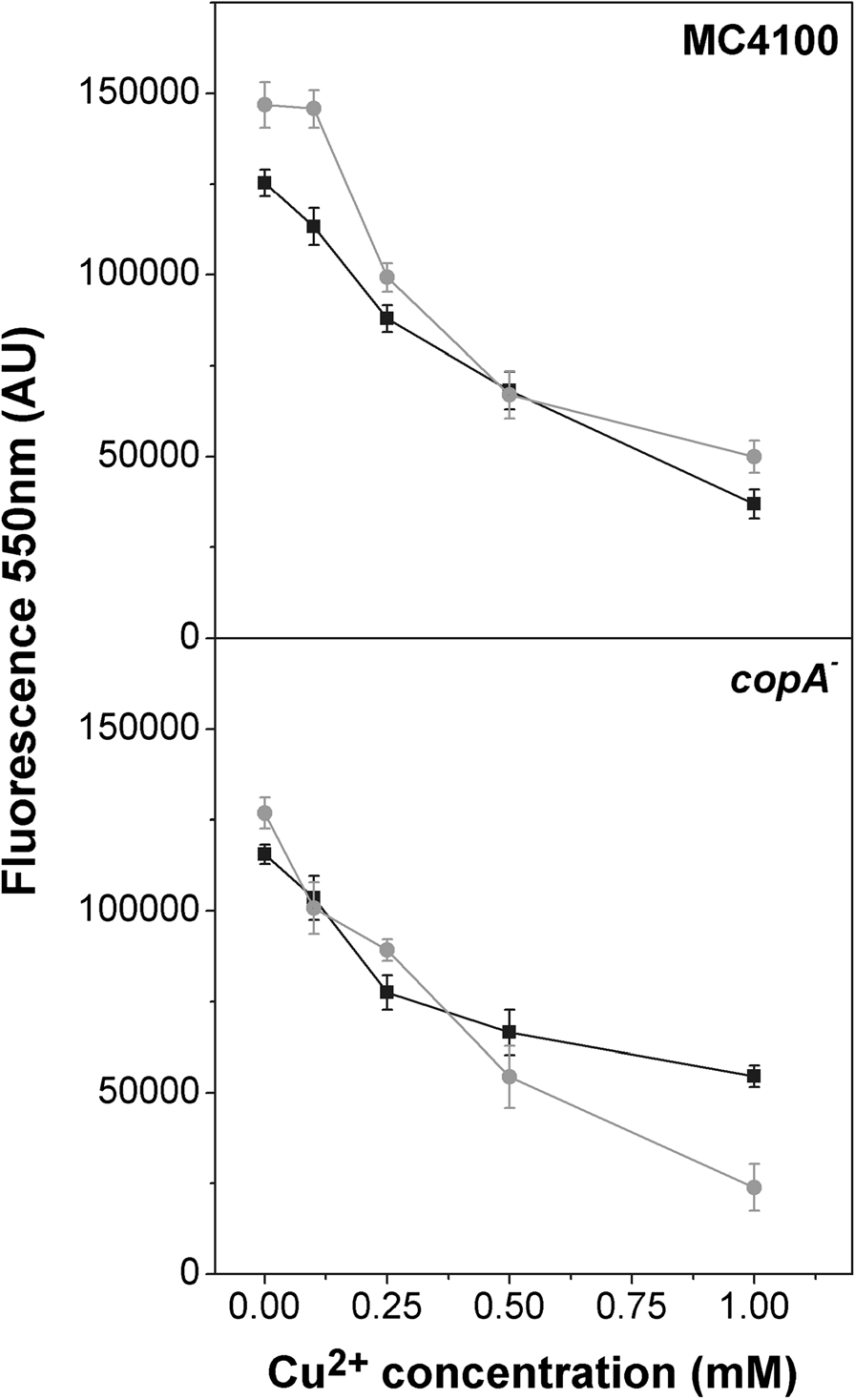
**PolyP levels of exponential phase cells exposed to copper.** Cells of the indicated strains grown in MT (**black line**) or MT + P (**grey line**) for 6 h were exposed with increase copper concentrations for 1 h. After incubation, polyP was quantified as described in Methods. Data are expressed as average ± SD of five independent experiments.

**Figure 7:**
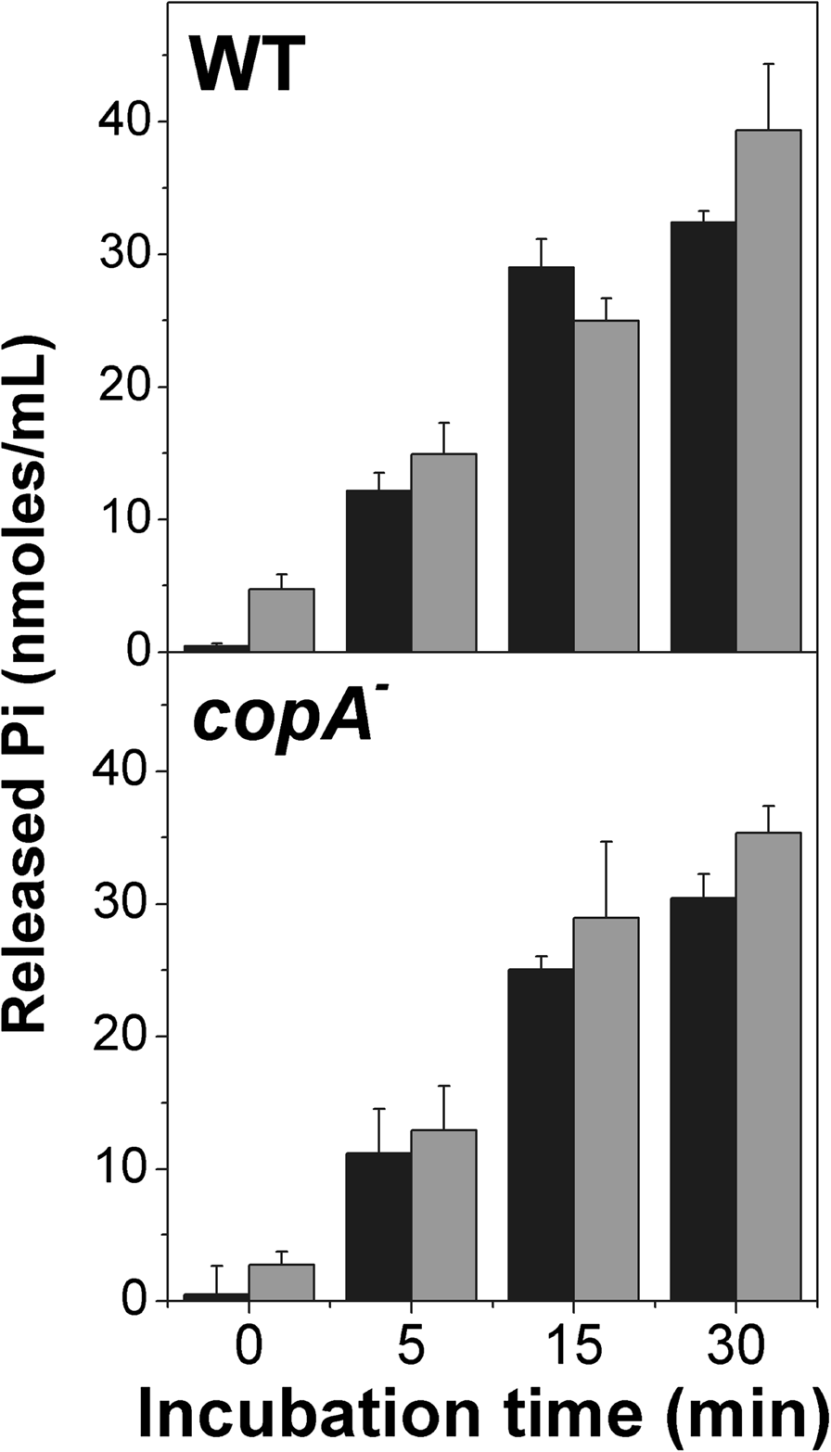
**Pi efflux from exponential phase cells exposed to copper.** 6 h MT (**black bars**) or MT + P (**grey bars**) cells of the indicated strains were resuspended in T buffer and exposed to 0.25 mM Cu^2+^ during different times. Pi was quantified in supernatants as described in Methods. Data are expressed as average ± SD of three independent experiments.

As shown above, polyP degradation and Pit system are involved in copper tolerance in stationary phase only in MT + P cells. Thus, we tested whether this detoxification mechanism is also feasible in exponential phase. During this phase, not only WT cells but also *ppx*^*−*^ and *ppk*^*−*^*ppx*^*−*^ mutants were tolerant to 0.5 mM Cu^2+^ even in MT (Figure 5A-C). PolyP degradation and Pi release were induced by copper exposure in WT cells grown in both media (Figures 6 and 7). These results are consistent with the presence of high intracellular polymer levels in WT cells at 6 h of growth, independently of media Pi concentration (Table 1). However, copper resistance of polyP metabolism lacking strains, indicates that another system is involved in Cu^2+^ tolerance during exponential phase. The involvement of CopA, a central component in *E. coli* copper detoxification during exponential phase [16], was evaluated in our experimental conditions using *copA*^*−*^, *copA*^*−*^*ppk*^*−*^*ppx*^*−*^*, copA*^*−*^*ppx*^*−*^ strains. *copA*^*−*^ cells were as resistant to copper as WT, while *copAppkppx* and *copAppx* mutants were highly sensitive to copper exposure (Figures 5D-F). As in WT, polyP degradation and Pi efflux occurred upon copper exposure in the *copA*^*−*^ background (Figures 6 and 7). Together, in order to tolerate copper in exponential phase, polyP-Pit system could be active to safeguard CopA absence or *vice versa*.

## Discussion

Cellular functions can be disrupted when Cu^2+^ concentration exceeds acceptable levels [27]. In order to survive the adverse environment, several mechanisms of resistance are switched on in bacteria [28]. In the present study, we demonstrated that polyP levels and Pit system are involved in *E. coli* copper tolerance.

In stationary phase, the significant metal resistance of WT cells grown in high phosphate medium could be attributed to the high polyP level in this condition [22], which could also account for enhancement in stationary-phase fitness [21]. The copper sensitivity of *ppk*^*−*^*ppx*^*−*^ is in agreement with previous work showing that this double mutant is deficient in stationary phase functions and lacks stress resistance [22,24,25]. On the other hand, considering *ppx* single mutant sensitive phenotype, not only polyP presence but also its degradation is relevant for Cu^2+^ resistance in our conditions, discarding the role of polymer merely as a metal chelator. The chelating effect constitutes one line of thought linking the metal tolerance and the polymer; however, abundance of polyP in exopolyphosphatase deficient strain may be damaging for the cell. Note that polymer molecules with high capacity to bind metal ions represent a source of potentially toxic species in equilibrium with the intracellular medium. Degradation of preformed polyP and Pi-copper complex formation that can be exported from the cells represent another alternative way to detoxify metals. In fact, our results provided lines of evidence that copper-induced polyP degradation through PPX in few minutes of exposure. In agreement, *Acidithiobacillus ferrooxidans* and *Sulfolobus metallicus* cells underwent to an increase of exopolyphosphatase activity with a concomitant decrease in polyP levels with increasing copper concentrations [8,9]. In addition, viability assays with Pit system mutants indicate, for the first time, the direct involvement of PitA and PitB in *E. coli* copper tolerance, as it was previously suggested for other metals [7] and copper [8,9]. Levels of *pitA* gene expression were invariant due to copper addition in each of our experimental conditions (data not shown). Expression was high in exponential phase either in MT and MT + P cells and moderate in stationary MT + P cells, coincidentally with copper tolerance conditions. In sufficient Pi medium MT, expression decay during stationary phase, where viability was impaired and polyP was minimal.

We consider that copper tolerance is a consequence of changes in polyP levels exerted by the metal. Even when copper efflux or formation of intracellular copper–phosphate complexes were not determined in this work, high Pi release and elevated membrane polarization in MT + P WT stationary phase cells, evidence that high polyP levels and its metal-induced degradation would lead to Cu^2+^-phosphate complexes formation and their subsequent efflux. Low changes in membrane polarization generated after copper addition in other strains and conditions may be due to differential diffusion of ions that induces complex movement of buffer and other ions.

According to present data and our previous results [21-23,29], the salt composition of the culture media should be carefully considered in the experimental design, especially when stationary-phase events are studied. Note that commonly used minimal media, as M63 [30] and M9 [31], contain Pi concentrations higher than 40 mM. Our strategy using differential Pi concentration media, allowed us to find the first copper detoxification mechanism acting in *E. coli* stationary phase, which only involves polyP-Pit system and is functional in high phosphate media. It should be noted that no copper induction of *copA* gene expression was observed in stationary phase in all the tested media (data not shown).

Our data show that polyP-Pit system is involved in copper tolerance also in exponential phase. Actually, CopA absence could be counteracted by a functional polyP-Pit system and, conversely, CopA would be responsible for metal tolerance in a polyP or Pit deficient background. Even we could not discard the participation of other copper detoxification mechanisms already described to be functional during this phase [17,19,28], CopA or polyP-Pit systems seem to be necessary to safeguard cells against copper toxicity, according to sensitive phenotypes of *copA*^*−*^*ppk*^*−*^*ppx*^*−*^ and *copA*^*−*^*ppx*^*−*^ strains. As it was previously described for *E. coli*[22], *Pseudomonas fluorescens*[32]*Corynebacterium glutamicum*[33]*, Bacillus cereus*[34] and a wide range of microorganisms [35], high polyP levels were reached in the early exponential growth phase. Thus, polyP-Pit system would be a very important aspect to consider as an additional copper tolerance mechanism in bacterial exponential phase.

## Conclusion

In conclusion, this work shed light on the previously proposed polyP-dependent mechanism for metal resistance in microorganisms. PolyP degradation and functionality of Pit, postulated as a metal-phosphate transporter system, mediates copper tolerance in *E. coli* both in exponential and stationary cells. Data represent the first experimental evidence of the involvement of Pit system components in this detoxification mechanism. Our study may also help to understand the importance of the environmental salt composition to regulate the microbial physiological state.

### Ethics Statement

The present research does not involve human subjects, human material, human data, or animals.

## Methods

### Strains and growth conditions

Bacterial strains are shown in Table 2. Mutations and *copA-lacZ* transcriptional fusion were transduced by P1 vir lysates into MC4100 strain. Cells were grown aerobically at 37°C with linear shaking in the saline minimal media, MT (2 mM phosphate) [36] and MT + P (defined as MT containing 40 mM phosphate buffer pH 7) [23]. Media were supplemented with 0.5% glycerol and 0.1% tryptone. Growth was monitored by measuring OD_560 nm_. When required, the following antibiotics were used: 100 μg mL^−1^ ampicillin, 30 μg mL^−1^ chloramphenicol and 50 μg mL^−1^ kanamycin.

**Table 2:** ***E. coli *** **strains and plasmids used in this work**

**Strains and plasmids**	**Relevant genotype or description**	**Construction or reference**
MC4100	*araD, lac, rpsL, flbB, deoC, ptsF, rbsR, relA1*	[37]
LSB022	MC4100 (*ppkppx*::Km)	[22]
LSB022/pBC29	LSB022/pBC29((*ppkppx::Km* /*ppk*^*+*^, Ap)	[29]
RKP2935	RKP4353 [Φ(*pitA–lacZ*)] *pitA::Cm*	[38]
AN3901	JC7623 *pitB::Cm*	[39]
AN4080	*pitA1 pitB*::Cm	[39]
LSB026	MC4100 *pitA:: Cm*	(P1(RKP2935)xMC4100)
MGP001	MC4100 *pitB::Cm*	(P1(AN3901)xMC4100)
JW0473-3	*F-, araD-araB, lacZ, copA::km λ*^*−*^*, rph-1, rhaD-rhaB, hsdR*	CGSC
MGP002	MC4100 *copA::Km*	(P1(JW0473-3)xMC4100)
MGP003	MGP002 *copA::FRT*	This study
MGP004	MGP003 *ppkppx::Km, copA*^*−*^	(P1(LSB022)xMGP003)
MGP005	MGP004 /pBC29 ((*ppkppx::Km*, *copA*^*−*^ /*ppk*^*+*^*, Ap*)	This study
pBC29	*(ppk*^*+*^*, Ap)*	[24]
pCP20	*Ap, Cm, cI857 lPR flp pSC101 oriTS*	[40]

### Cu^2+^ tolerance determination

Cells grown in MT and MT + P during 6, 24 or 48 h were incubated with shaking at 37°C for 1 h with different CuSO_4_ concentrations in the same culture media. Identical aliquots of cells incubated without copper were used as controls. Then, metal tolerance was evaluated by qualitative viability assays, spotting 1/10 serial dilutions on LB-agar [21]. Plates were incubated for 24 h at 37°C.

### PolyP level measurement

Intracellular polyP was measured in cell suspensions by a fluorescence approach using 4′,6-diamidino-2-phenylindole (DAPI) [41]. Briefly, cells were washed and resuspended in T buffer (100 mM Tris–HCl, pH 8). 17 μM DAPI (Sigma) was added to cuvettes containing cell suspensions (OD_560 nm_ =0.02) in T buffer, with 0.075% SDS and chloroform for cell permeabilization [29]. After 5 min at 37°C with agitation, the DAPI fluorescence spectra (excitation, 415 nm; emission, 445–650 nm) were recorded using an ISS PCI spectrofluorometer (ISS Inc., Champaign, IL). Fluorescence of the DAPI-polyP complex at 550 nm was used as a measurement of intracellular polyP, since emissions from free DAPI and DAPI-DNA are minimal at this wavelength [41].

### Membrane electrical potential measurement

Changes in the transmembrane electrical potential (ΔΨ) were measured utilizing the potential sensitive fluorescent probe 3,3′-dipropylthiadicarbocyanine (DisC3 [5]) [42]. Briefly, cells were harvested by centrifugation, washed twice with 100 mM T buffer and resuspended in 5 mM HEPES buffer pH 7.5 to an OD _560 nm_ = 0.1. Cell suspensions were incubated with shaking plus 0.4 μM DisC3 [5] and 0.4% glucose. Fluorescence measurements were carried out at 37°C, adjusting the wavelengths of excitation and emission to 622 and 675 nm, respectively. When the dye uptake was maximal, as indicated by a decrease to a steady fluorescence value, (ΔΨ_i_), 0.1 mM Cu^2+^ was added and fluorescence was followed for 5 min, achieving ΔΨ_f_. The difference between ΔΨ_f_ and ΔΨ_i_ was defined as ΔΨ_Cu_. Measurements were repeated at least seven times under each condition. Distillated water was added instead of Cu^2+^ solutions in negative control.

### Pi efflux determination

Cells were harvested and thoroughly washed by four steps of centrifugation and resuspension with T buffer to eliminate Pi present in the media. Then, cells were resuspended to the original volume in the same buffer (OD between 2.5 to 3, corresponding to ≈ 10^9^ CFU mL^−1^) and incubated with agitation in the presence of 0.25 mM Cu^2+^ at 37°C for the indicated times. Phosphate was determinate in supernatants using ammonium molybdate and ascorbic acid as described by Chifflet *et al*. [43]. T buffer incubated with copper for 60 min and cells without metal were used as negative controls.

### Gene expression

Gene expression was evaluated by β-galactosidase activity and expressed in Miller Units (MU) [44].

### Statistical analysis

Data were subjected to analysis of variance (ANOVA) followed by Tukey’s test with Statitix 9.0 Analytical Software 2008 for Windows (USA). Differences at *p-*value of 0.05 were considered significant.

## Competing interests

The authors declare that they have no competing interests.

## Authors’ contributions

MGP carried out determinations of Cu^2+^ tolerance, polyP level, membrane electrical potential, Pi efflux, and gene expression. LASB initiated experiments in Cu^2+^ tolerance and polyP level measurements. MGP and LRM performed statistical analysis. MGP, MRR, and VAR prepared the manuscript and participated in the analysis of data. All authors designed the study and revised the manuscript for intellectual content. All authors read and approved the final manuscript.
